# Is anti‐snake venom required for all snakebites: A case report

**DOI:** 10.1002/ccr3.2598

**Published:** 2019-12-08

**Authors:** Thinley Dorji

**Affiliations:** ^1^ Samtse General Hospital Samtse Bhutan

**Keywords:** 20‐minute WBCT, anti‐snake venom, snakebite, whole blood clotting time

## Abstract

The classification of snakebite is based on clinical examination and laboratory tests. In cases, of suspected hemotoxic snakebite, the anti‐snakebite venom (ASV) is administered based on 20‐minute whole blood clotting time. However, the use of ASV should be guided by the presence of bleeding diathesis along with raised clotting time.

## Graphical abstract

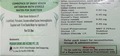

## 1 INTRODUCTION

The most common diagnostic method for hemotoxic snakebite is 20‐minute whole blood clotting time. The management of hemotoxic snakebite requires administration of anti‐snake venom to patients with prolonged CT or bleeding disorders until CT returns to normal. We report two case reports where the clotting time turned to normal.

Snakebite is a neglected public health problem, commonly among countries in temperate and tropical climate.^1^ They are reported globally but majority of them occur in Asia, Africa, and Latin America.^2^ Of this, the highest occur in the South‐East Asia.^3^ The snakebites are commonly seen among farmers and children.^2^, ^4^

Asia is home to an estimated 300 varieties of snakes including at least 67 varieties of Elapidae (cobras and kraits) and Viperidae (pit viper, Russel viper, and green pit viper).^4^ Both groups can present with local signs at bite area with pain, local swelling, and blisters. However, Viperidae present with ecchymosis, renal failure, pulmonary edema, and bleeding. Envenomation by Elapidae group and Russel viper presents with neurological signs such as altered mental status, cranial nerve involvement, and peripheral motor weakness to paralysis.^1^, ^5^ In South‐East Asia, the clotting disorders following snakebite is diagnostic of viper bite.^5^

The common method of diagnosis of venomous snakebite is usually done clinically. Coagulation studies like prothrombin time, and renal and hepatic function test can aid in the diagnosis of venomous snakebite. However, in most developing countries with resource constraints, the diagnosis of envenomation is usually based on 20‐minute whole blood clotting time (20WBCT) at the bed site.^5^, ^6^ In this test, 2 mL of blood is collected in a clean glass container and left undisturbed at ambient temperature for 20 minutes. The container is tipped to check for coagulation. The absence of clot is suggestive of venomous snakebite.^1^, ^2^

Currently, the anti‐snake venom is administered to patients with suspected snakebite with signs of systemic envenomation (spontaneous bleeding, prolonged clotting time, neurotoxic signs, acute kidney injury) or local swelling involving more than half of limbs. The anti‐snakebite venom should be given immediately and repeatedly until the 20WBCT returns to normal.^5^ However, the administration of ASV is not without risks. More than 10% of the patients who receive ASV develop some reaction varying from mild urticarial rash to severe anaphylactic reaction.^2^ These call for thorough examination of patients before the administration of ASV in snakebite cases. Moreover, inappropriate usage can also lead to shortage of this scarce, relatively costly antidote.

Here we report two cases, where patients received two doses of anti‐snake venom. However, ASV was discontinued in spite of high 20WBCT since patient was clinically normal without any bleeding diathesis. The 20WBCT returned to normal after few days.

## 2 CASE STUDY

**Figure 1:**
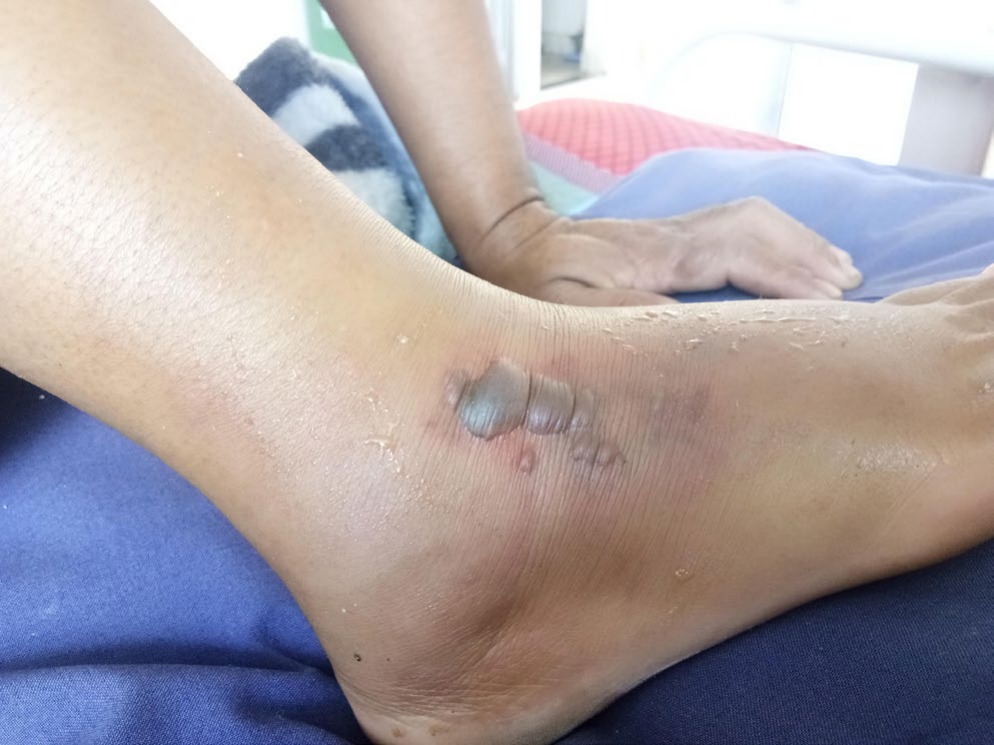
Blister formation at the site of bite on leg

1. Eighteen‐year‐old female student was brought to the emergency ward of the hospital with history of snakebite to her right foot while walking to her school hostel from dining hall in the evening. At the time of bite, there was mild bleeding from the site which stopped spontaneously. She then tied a tourniquet above her ankle with a piece of cloth, after which the swelling started. She was initially taken to the nearby health center where she received a dose of tetanus vaccine. On admission, her blood pressure was 120/80 mm Hg, pulse rate was 90/minutes, temperature was 38°C, and oxygen saturation was 98% with room air. On examination, there were blisters at the site of bite with signs of cellulitis (Figure 1). She did not have any neurological weakness. The hematological examination showed total leukocyte count at 11 100/mm^3^, hemoglobin at 11.9 gm/dL but with prolonged 20‐min WBCT. The renal and liver function tests were within normal range.

Due to the presence of coagulopathy, she was confirmed to have been bitten by venomous snake. She was immediately given ten vials of anti‐snake venom (ASV) along with injection 500 mg ampicillin QID and tablet cloxacillin 500 mg QID on admission. The 20‐minute WBCT was repeated after six hours and still found to be prolonged. She was given an additional dose of 10 vials of ASV. She had no bleeding from the wound or any other orifices. The swelling in her leg remained the same as at the time of admission. The repeat 20WBCT remained prolonged in spite of two doses of ASV. Since the patient did not have any bleeding diathesis, no additional ASV was given. The patient's blood pressure, pulse rate, and oxygen saturation were closely monitored along with any bleeding from bite site, gum bleeding, and gastrointestinal bleeding. The clotting time and her liver function test were monitored on daily basis.

The blisters on her right foot ruptured subsequently and wound got infected. Daily dressing was done, and daily monitoring of her clotting time was done. The patient was finally discharged on 12th day when her 20WBCT time dropped to 15 minutes.

- Eight‐year‐old female student was brought to the hospital by her mother with complaint of snakebite to her right foot two days back while walking back home from school in the evening. She had pain at the site of bite which subsided spontaneously. She complained of mild bleeding from bite site which stopped after she washed the wound with soap and water. There was no history of coagulopathy in her family.

**Figure 2:**
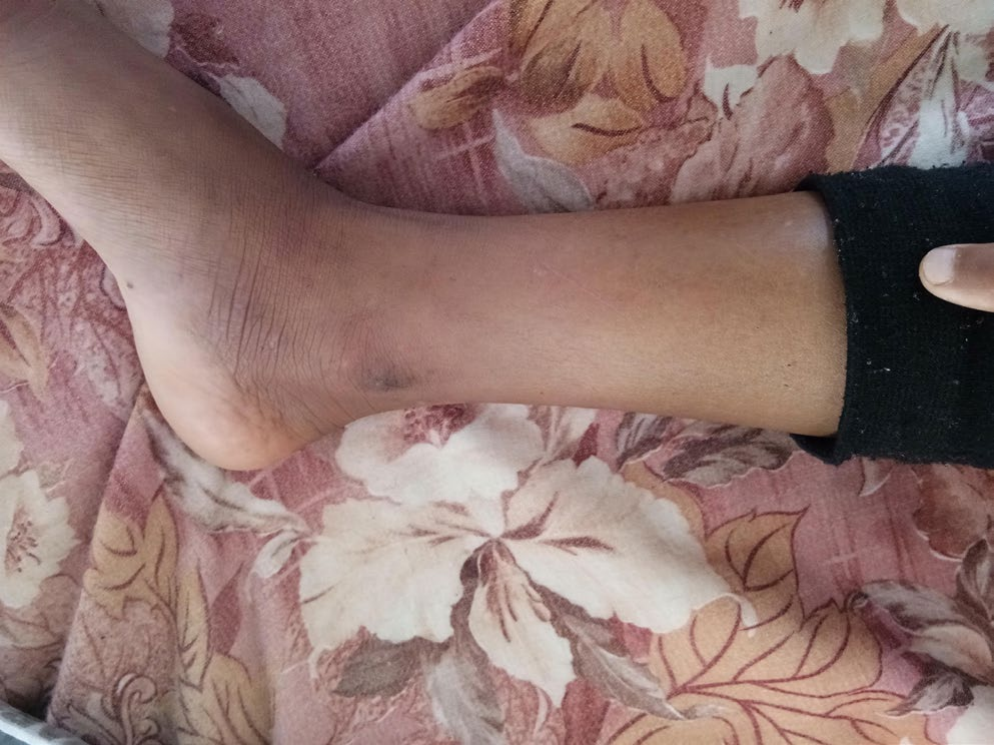
Site of bite

On examination, her BP was 110/60 mm Hg, pulse rate of 84/minutes, body temperature of 37.5°C, and SpO_2_ of 98% with room air. The bite mark was visible on her right foot (Figure 2). On admission, her WBCT was more than 20 minutes. Her total leukocyte count was 12 000/mm^3^, and hemoglobin was 11.6 gm/dL with platelet of 421 000/mm^3^. The liver and renal function tests were within normal range. The patient received 10 vials of ASV. The 20‐minutes WBCT was evaluated after 6 hours again which was found to be high. Therefore, an additional 10 vials of ASV were given.

The 20‐minutes WBCT was repeated next day and was still found to be high. Since the patient did not have any bleeding disorders, she was not given additional doses of ASV. The patient's vitals, clotting time, and liver function test were monitored on daily basis. During all these days, she was asymptomatic and had no bleeding diathesis. Her WBCT dropped to <20 minutes after one week of admission and was subsequently discharged.

## 3 DISCUSSION

**Figure 3:**
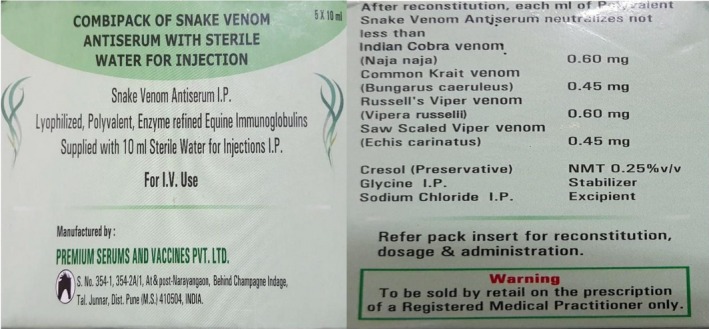
Picture of antivenom used in Bhutan

Snakebite is a common medical problem in Bhutan especially in southern belt which lies in subtropical areas. It has the highest proportion of snakebite in South‐East Asia at 4.13 per 1000 population.^7^ In 2017, 208 cases of snakebites were reported from health centers across the country.^8^ At least 84 types of snakes have been identified in Bhutan.^9^ However, the majority of snakebites in Bhutan are nonvenomous.^10^ The venomous snakes include cobra, krait, and viper species.^11^ Of the hemotoxic snakebites, viper bite is the most common.^12^ Therefore, the above two cases were assumed to be due to viper bites due to the presence of coagulopathy. ASV available in Bhutan is polyvalent manufactured in India. It protects against four common species (Russell's viper, cobra, Krait, and saw‐scaled viper) (Figure 3).

World Health Organization recommends administration of ASV to all patients with signs of local or systematic envenomation. The repeat dose of ASV is guided by clinical improvement and 20‐minutes WBCT which are repeated every six hours since liver takes at least six hours to produce clotting factors. Therefore, ASV has to be given to all patients with prolonged CT and the dose repeated every six hourly until the 20WBCT becomes normal.^5^, ^13^

In the above two‐case series, the patient's coagulopathy did not improve even after administration of two doses of ASV. However, the patient did not have any signs of bleeding and all blood works were within normal limit. The clotting time improved spontaneously after a week. The reason behind this is unknown. However, it could be due to low dose of venom during the bite. This calls for further prospective trials to determine the indications of ASV especially among those snakebite patients with stable vitals but with prolonged clotting time.

The ASV is usually expensive and not easily available. Inappropriate and irrational use of it can lead to hypersensitivity reactions and deaths. Therefore, for the management of snakebite cases, risk of bleeding also should be considered apart from clinical and laboratory parameters.

## 4 CONCLUSION

The ASV is administered for all snakebite patients with signs of envenomation based on clinical examination findings or raised 20‐minutes WBCT. However, the administration of ASV can be delayed if patient is clinically normal with no organ involvement even with raised clotting time. Further studies are needed to confirm this finding.

## CONFLICT OF INTEREST

The author has no potential conflict of interest to declare.

Consent: Consent was taken from both the patient as well as the parents for taking photograph and writing of this case report.

## AUTHOR'S CONTRIBUTION

TD: performed the clinical diagnosis, conceived the idea, drafted the manuscript, and submitted the final revision of the manuscript for publication.
